# lifex-fiber: an open tool for myofibers generation in cardiac computational models

**DOI:** 10.1186/s12859-023-05260-w

**Published:** 2023-04-12

**Authors:** Pasquale Claudio Africa, Roberto Piersanti, Marco Fedele, Luca Dede’, Alfio Quarteroni

**Affiliations:** 1 MOX, Department of Mathematics, Politecnico di Milano, Milano, Italy; 2 Institute of Mathematics, École Polytechnique Fédérale de Lausanne, Lausanne, Switzerland

**Keywords:** Computational cardiology, High-performance computing, Cardiac fibers, Mathematical modeling, Finite element methods, Primary 68-04, 68N30, Secondary 35-04, 65-04, 65M60, 65N30, 65Y05, 65Y20, 92-04, 92C50

## Abstract

**Background:**

Modeling the whole cardiac function involves the solution of several complex multi-physics and multi-scale models that are highly computationally demanding, which call for simpler yet accurate, high-performance computational tools. Despite the efforts made by several research groups, no software for whole-heart fully-coupled cardiac simulations in the scientific community has reached full maturity yet.

**Results:**

In this work we present $\texttt {life}^{\texttt {x}}$-fiber, an innovative tool for the generation of myocardial fibers based on Laplace-Dirichlet Rule-Based Methods, which are the essential building blocks for modeling the electrophysiological, mechanical and electromechanical cardiac function, from single-chamber to whole-heart simulations. $\texttt {life}^{\texttt {x}}$-fiber is the first publicly released module for cardiac simulations based on $\texttt {life}^{\texttt {x}}$, an open-source, high-performance Finite Element solver for multi-physics, multi-scale and multi-domain problems developed in the framework of the iHEART project, which aims at making *in silico* experiments easily reproducible and accessible to a wide community of users, including those with a background in medicine or bio-engineering.

**Conclusions:**

The tool presented in this document is intended to provide the scientific community with a computational tool that incorporates general state of the art models and solvers for simulating the cardiac function within a high-performance framework that exposes a user- and developer-friendly interface. This report comes with an extensive technical and mathematical documentation to welcome new users to the core structure of $\texttt {life}^{\texttt {x}}$-fiber and to provide them with a possible approach to include the generated cardiac fibers into more sophisticated computational pipelines. In the near future, more modules will be successively published either as pre-compiled binaries for x86-64 Linux systems or as open source software.

## Background

The human heart function is a complex system involving interacting processes at the molecular, cellular, tissue, and organ levels with widely varying time scales. For this reason, it is still among the most arduous modeling and computational challenges in a field where *in silico* models and experiments are essential to reproduce both physiological and pathological behaviors [1].

A satisfactorily accurate model for the whole cardiac function must be able to describe a wide range of different processes, such as: the propagation of the trans-membrane potential and the flow of ionic species in the myocardium, the deformation caused by the muscle contraction, the dynamics of the blood flow through the heart chambers and cardiac valves [2]. In particular, the dynamics of ionic species needs for accurate models specifically designed to reproduce physiological [3] and pathological scenarios [4, 5] (such as the *ten Tusscher–Panfilov* [6] and the *Courtemanche-Ramirez-Nattel* [7] ionic models for ventricular/atrial cells, respectively).

These demanding aspects make whole-heart fully-coupled simulations computationally intensive and call for simpler yet accurate, high-performance computational tools.

In this work we introduce $\texttt {life}^{\texttt {x}}$-fiber, an innovative tool for the generation of myocardial fibers based on $\texttt {life}^{\texttt {x}}$ [8], an open-source, high-performance Finite Element (FE) numerical solver for multi-physics, multi-scale and multi-domain differential problems. It is written in C++ using the most modern programming techniques available in the C++17 standard and is built upon the deal.II^1^ [9] FE core. The code is natively parallel and designed to run on diverse architectures, ranging from laptop computers to High Performance Computing (HPC) facilities and cloud platforms. We tested our software on a cluster node endowed with 192 cores based on Intel Xeon Gold 6238R, 2.20 GHz, available at MOX, Dipartimento di Matematica, Politecnico di Milano, and on the GALILEO100 supercomputer available at CINECA (Intel CascadeLake 8260, 2.40GHz, see https://wiki.u-gov.it/confluence/display/SCAIUS/UG3.3%3A+GALILEO100+UserGuide for more technical specifications).

Despite being conceived as an academic research library in the framework of the iHEART project (see Section “Funding”), $\texttt {life}^{\texttt {x}}$ is intended to provide the scientific community with a Finite Element solver for real world applications that boosts the user and developer experience without sacrificing its computational efficiency and generality.

**Fig. 1:**
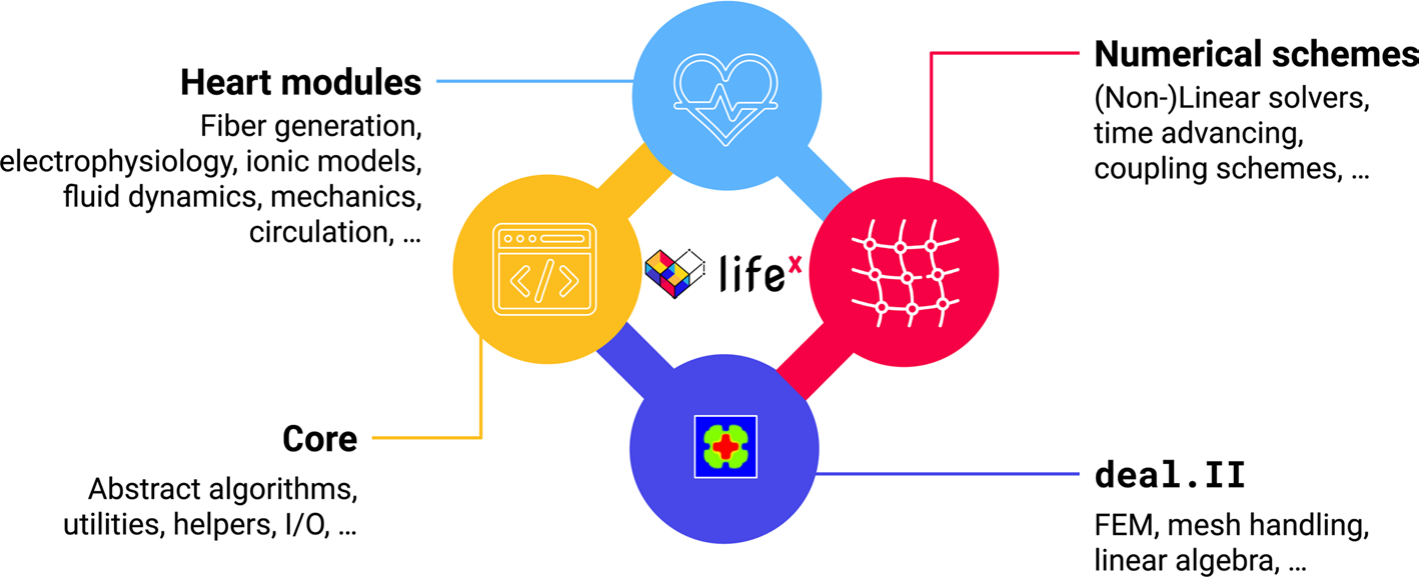
$\texttt {life}^{\texttt {x}}$ modules: the library provides core functionalities and a framework for the numerical solution of the Finite Element problems described in Section “Laplace-Dirichlet rule-based methods”. $\texttt {life}^{\texttt {x}}$-fiber is the first publicly released *heart module* based on $\texttt {life}^{\texttt {x}}$

Since its initial development, $\texttt {life}^{\texttt {x}}$ has served as the core block to build several modules for the simulation of the cardiac function, such as electrophysiology, mechanics, electromechanics, blood fluid dynamics, and myocardial perfusion [8], as displayed in Fig. 1. Such models have been recently exploited for a variety of standalone or coupled simulations both under physiological and pathological conditions (see, e.g., [4, 5, 10–17]).

As the present release focuses on modeling the cardiac fibers, in the next two paragraphs we will briefly review the physiology of myofibers and the most used mathematical methods to model them, describing in detail their implementation which is included within $\texttt {life}^{\texttt {x}}$-fiber.

### Cardiac fibers: physiology and modeling

The heart is a four chambers muscular organ whose function is to pump the blood throughout the whole circulatory system. The upper chambers, the right and left atria, receive incoming blood. The lower chambers, the right and left ventricles, pump blood out of the heart and are more muscular than atria. The left heart (i.e. left atrium and left ventricle) pumps the oxygenated blood through the systemic circulation, meanwhile the right heart (i.e. right atrium and right ventricle) recycles the deoxygenated blood through the pulmonary circulation [1, 2]. The atria and the ventricles are separated by the atrioventricular valves (mitral and tricuspid valves) that regulate the blood transfer from the upper to lower cavities. The four chambers are connected to the circulatory system: the ventricles with the aorta through the aortic valve and pulmonary artery via the pulmonary valve; the left atrium with the left and right pulmonary veins, whereas the right atrium with superior and inferior caval veins [1, 2].

The heart wall is made up of three layers: the internal thin *endocardium*, the external thin *epicardium* and the thick muscular cardiac tissue, the *myocardium*. Most of the myocardium is occupied by *cardiomyocytes*, striated excitable muscle cells that are joined together in linear arrays. The result of cluster cardiomyocytes, locally organized as composite laminar sheets, defines the orientation of muscular *fibers* (also called *myofibers*). Aggregations of myofibers give rise to the fiber-reinforced heart structure defining the cardiac muscular architecture [18, 19].

**Fig. 2:**
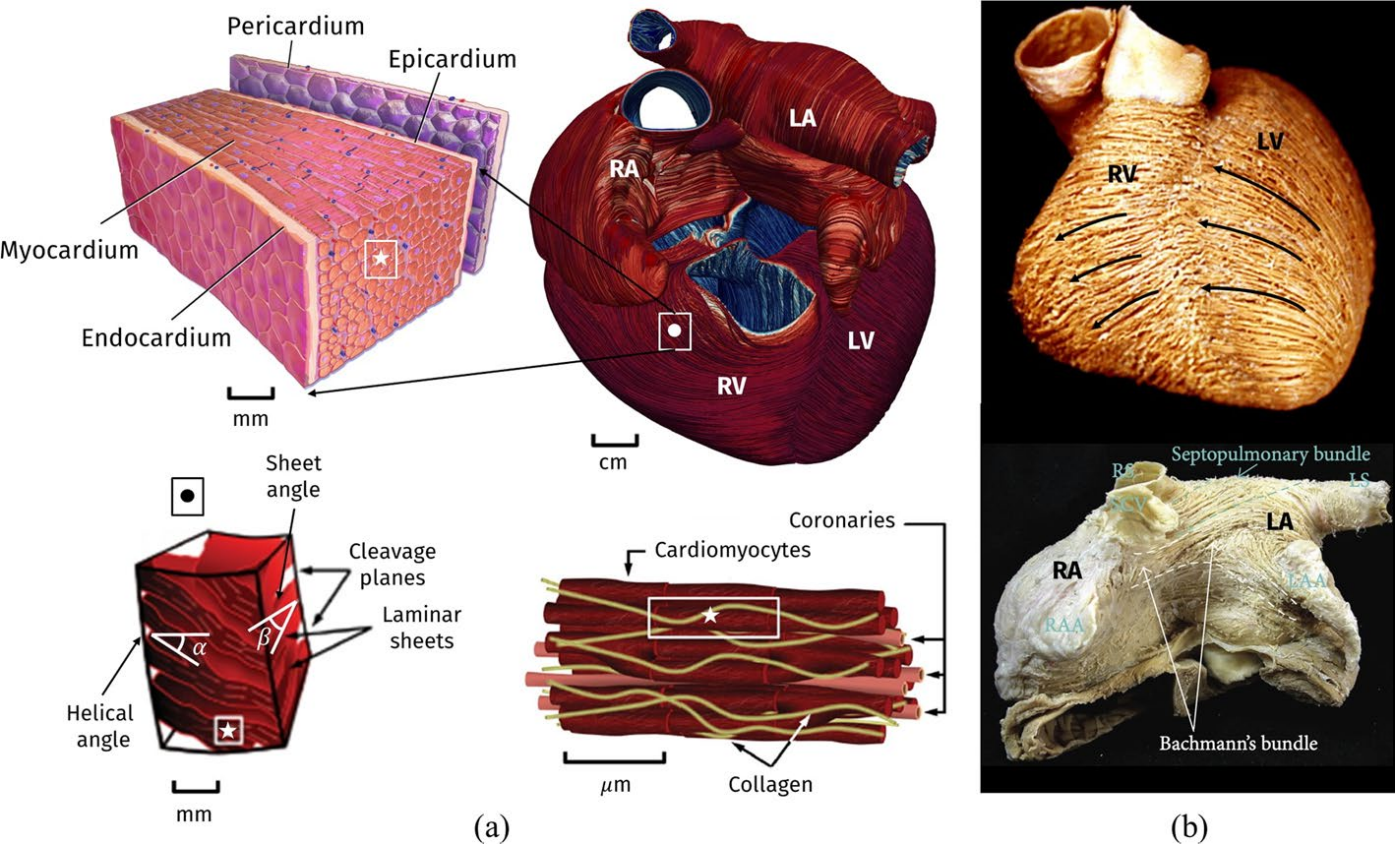
**a** Representation of the multiscale cardiac muscle. **b** Anatomical dissection of myocardial fibers in ventricles (top) and atria (bottom). Images taken and readapted from [54–57]. Images were available either freely under a Creative Commons Attribution license or have been granted reuse permission by the copyright holder

A schematic representation of the multiscale myocardial fiber-structure is shown in Fig. 2. Ventricular muscular fibers are well-organized as two intertwined spirals wrapping the heart around, defining the characteristic myocardial helical structure [20, 21]. Local orientation of myofibers is identified by their angle on the tangent plane and on the normal plane of the heart, called the *helical* and the *sheet* angles, respectively [18, 22]. The transition inside the myocardial wall is characterized by a continuous, almost linear change in helical angle from about $60^\circ$ at the epicardium to nearly $-60^\circ$ at the endocardium [18, 21].

Atrial fibers architecture is very different from that of the ventricles, where myofibers are aligned in a regular pattern [18]. Indeed, myofibers in the atria are arranged in individual bundles running along different directions throughout the wall chambers [23, 24]. Preferred orientation of myofibers in the human atria is characterized by multiple overlapping structures, which promote the formation of separate attached bundles [25], as shown in Fig. 2.

The cardiac muscular fiber architecture is the backbone of a proper pumping function and has a strong influence on the electric signal propagation throughout the myocardium and also on the mechanical contraction of the muscle [26–29]. This motivates the need to accurately include fibers orientation in cardiac computational models in order to obtain physically sound results [3, 17, 30].

Due to the difficulty of reconstructing cardiac fibers from medical imaging, different methodologies have been proposed to provide a realistic surrogate of myofibers orientation [21, 31–39]. Among these, atlas-based methods map and project a detailed fiber field, previously reconstructed on an atlas, on the geometry of interest, exploiting imaging or histological data [21, 34, 39]. However, these methods require complex registration algorithms and their results depend on the original atlas data upon which they have been built.

Alternative strategies for generating myofiber orientations are the Rule-Based Methods (RBMs) [3, 32, 35–37, 40–42]. RBMs describe fiber orientations with mathematically sound rules based on histological and imaging observations and only require information about the myocardial geometry [18]. These methods parametrize the transmural and apico-basal directions in the entire myocardium in order to assign orthotropic (longitudinal, transversal and normal) myofibers [3].

A particular class of RBMs, which relies on the solution of Laplace boundary-value problems, is known as Laplace-Dirichlet Rule-Based Methods (LDRBMs), addressed in [31–33] and recently analyzed under a unified mathematical formulation [3]. LDRBMs define the transmural and apico-basal directions by taking the gradient of harmonic functions (the potentials) corresponding to suitable Dirichlet boundary conditions. These directions are then properly rotated to match histological observations [18, 20, 21, 23].

This initial release includes a generator for myocardial fibers based on LDRBMs [3], with application to a number of different prototypical and realistic geometries (slab models, left ventricles and left atria).

### Laplace-Dirichlet rule-based methods

In this section, we briefly recall the LDRBMs that stand behind the myocardial fiber generation. In Section “Results and discussion” we will present several examples where we elaborate on how to reproduce and run the algorithms presented hereafter.

This *getting started* guide presents LDRBMs for (ventricular and spherical) slabs, (based and complete) left ventricular and left atrial geometries. For further details about the LDRBMs presented here see also [3].

The following common steps are the building blocks of all LDRBMs.

**Fig. 3:**
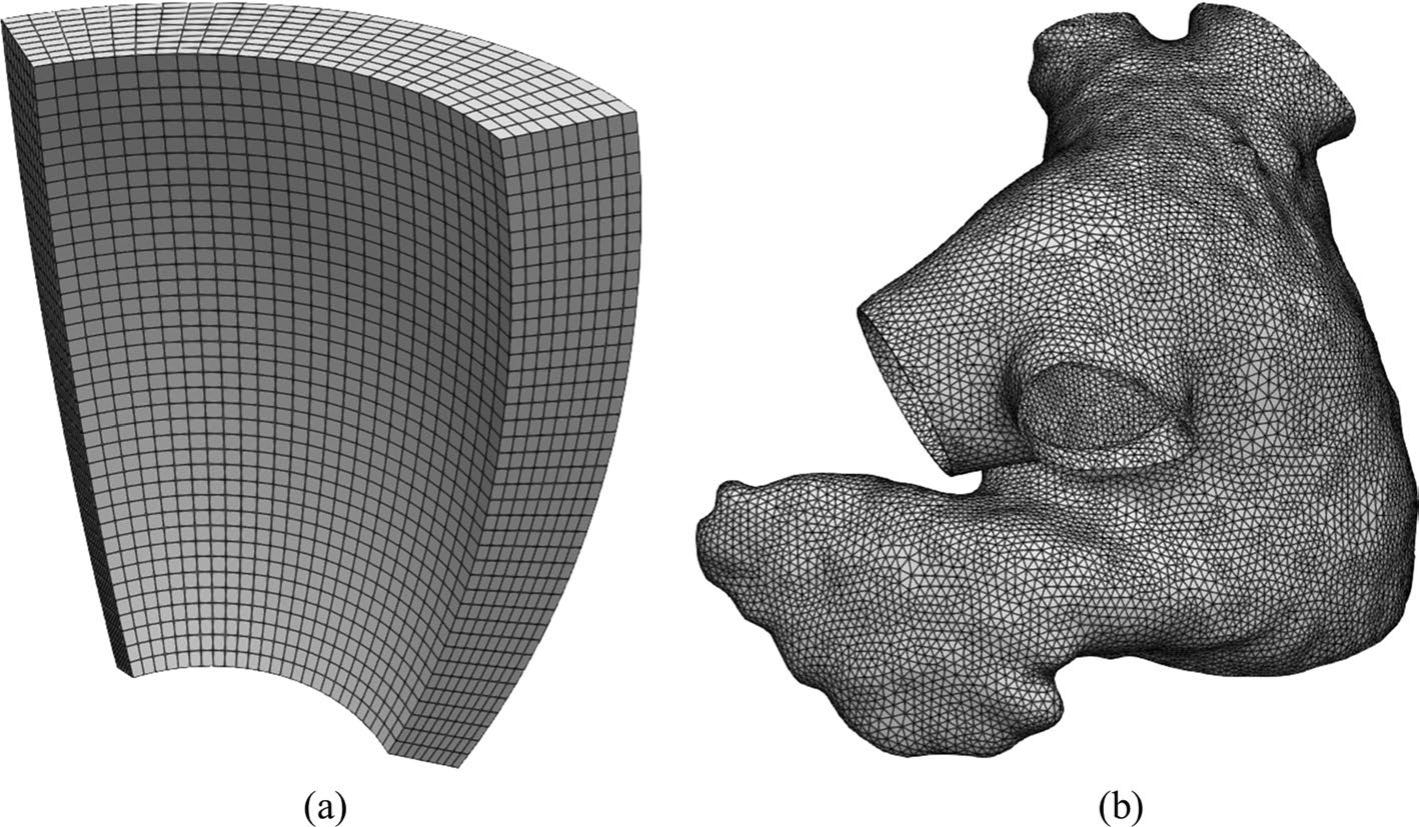
**a** Hexahedral mesh of a ventricular slab. **b** Tetrahedral mesh of a realistic left atrium [38]

1. Labeled mesh: A labeled volumetric mesh of the domain $\Omega$ must be provided to define specific partition of the boundary $\partial \Omega$ as
  $$\begin{aligned} \partial \Omega = \Gamma _\text{epi} \cup \Gamma _\text{endo} \cup \Gamma _\text{base} \cup \Gamma _\text{apex}, \end{aligned}$$
  where $\Gamma _\text{endo}$ is the endocardium, $\Gamma _\text{epi}$ is the epicardium, $\Gamma _\text{base}$ is the basal plane and $\Gamma _\text{apex}$ is the apex, which are demarcated through proper surface labels included in the input mesh. $\texttt {life}^{\texttt {x}}$ is designed to support both hexahedral and tetrahedral labeled meshes in the widely used *.msh format [8], see Fig. 3. This type of mesh can be generated by a variety of mesh generation software (e.g. gmsh,^2^netgen,^3^vmtk^4^ and meshtools^5^). Otherwise, other mesh-format types can be converted in *.msh using for example the open-source library meshio.^6^

**Fig. 4:**
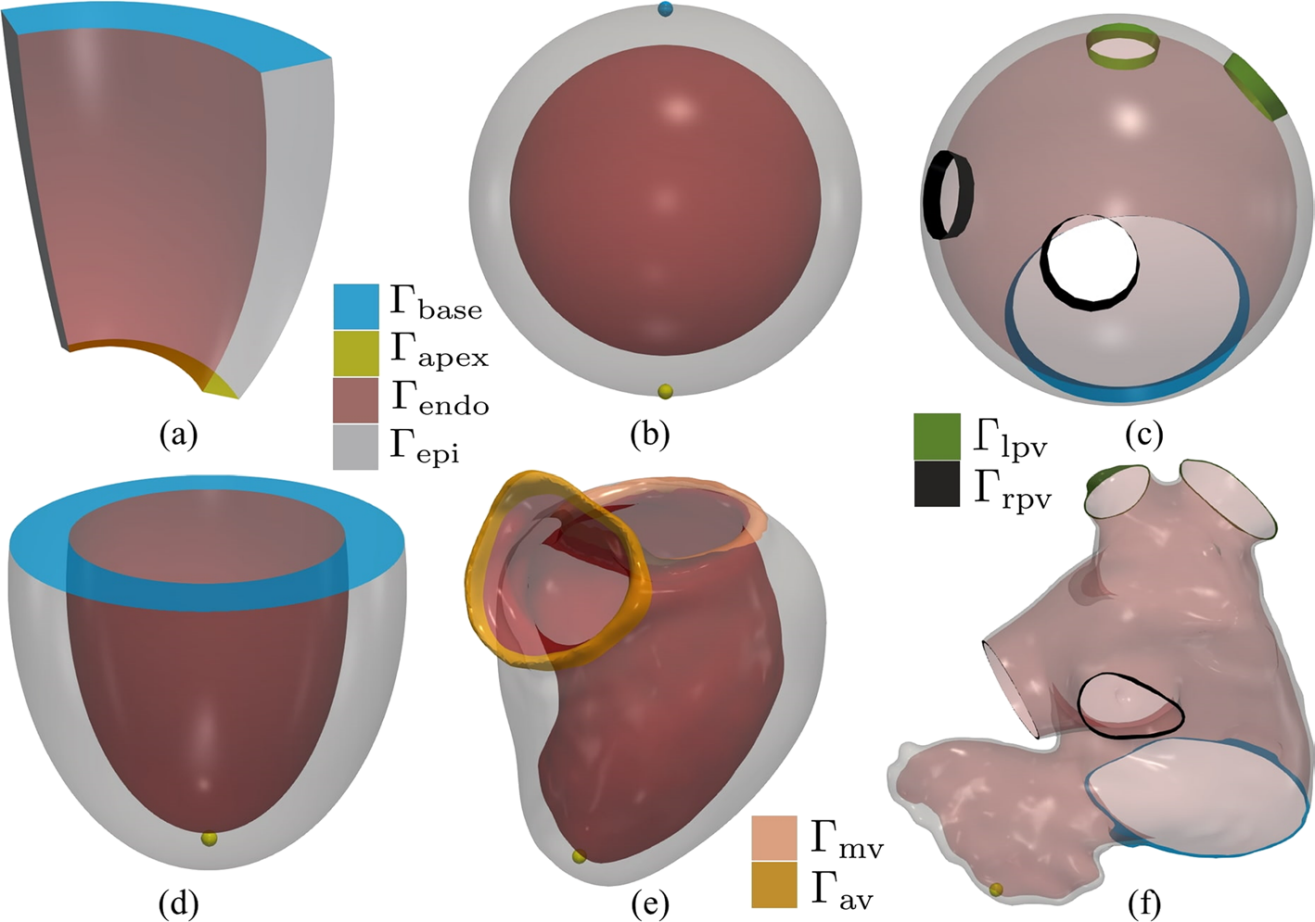
Labeled meshes. **a** Ventricular slab. **b** Spherical slab. **c** Idealized left atrium. **d** Idealized based left ventricle. **e** Realistic complete left ventricle. **f** Realistic left atrium. $\Gamma _\text{base}$ denotes the basal plane, $\Gamma _\text{apex}$ the apex, $\Gamma _\text{endo}$ the endocardium, $\Gamma _\text{epi}$ the epicardium, $\Gamma _\text{lpv}$, $\Gamma _\text{rpv}$ the left (right) pulmonary veins, respectively

Epicardium and endocardium: for the ventricular slab geometry $\Gamma _\text{endo}$ and $\Gamma _\text{epi}$ are the lateral walls of the slab, see Fig. 4a; for the spherical slab, the ventricular and atrial geometries $\Gamma _\text{endo}$ and $\Gamma _\text{epi}$ are the internal and external surfaces, see Fig. 4b–f.
Basal plane and apex: for the ventricular slab geometry $\Gamma _\text{base}$ and $\Gamma _\text{apex}$ are the top and bottom surfaces, respectively (see Fig. 4a); for the spherical slab, $\Gamma _\text{base}$ and $\Gamma _\text{apex}$ are selected as the north and south pole points of the epicardial sphere (see Fig. 4b); for the based ventricular geometry $\Gamma _\text{base}$ is an artificial basal plane located well below the cardiac valves (see Fig. 4d), whereas for the complete ventricular geometry $\Gamma _\text{base}$ is split into $\Gamma _\text{mv}$ and $\Gamma _\text{av}$, representing the mitral and aortic valve rings, respectively (see Fig. 4e); for the ventricular geometries $\Gamma _\text{apex}$ is selected as the epicardial point furthest from the ventricular base (see Fig. 4d, e); for the atrial geometry $\Gamma _\text{base}$ is the mitral valve ring and $\Gamma _\text{apex}$ represents the apex of the left atrial appendage (see Fig. 4c–f);
Atrial pulmonary rings: the atrial geometry type also requires the definition of the boundary labels for the left $\Gamma _\text{lpv}$ and right $\Gamma _\text{rpv}$ pulmonary vein rings, see Fig. 4c–f.

**Fig. 5:**
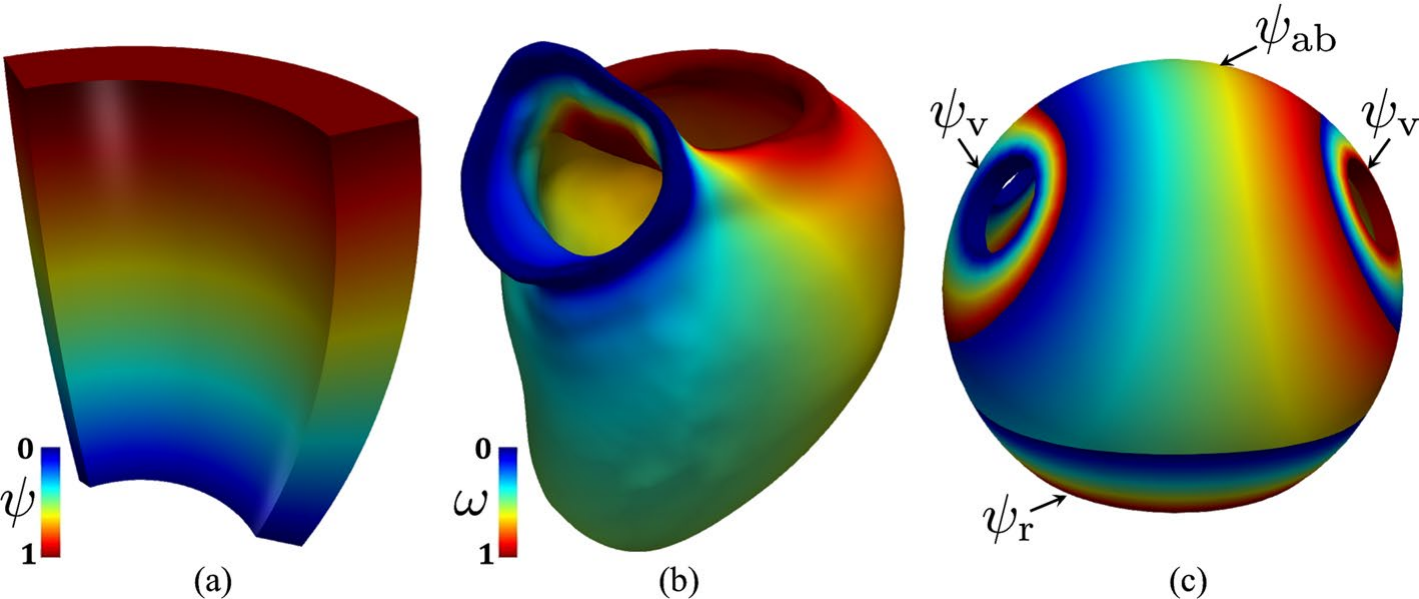
Different types of normal distances: **a** Bayer-Trayanova et al. approach [32]. **b** Doste et al. approach [33]. (c) Piersanti et al. approach [3]

2. Transmural direction: A transmural distance $\phi$ is defined to compute the distance of the epicardium from the endocardium, by means of the following Laplace-Dirichlet (LD) problem:
  1 $$\begin{aligned} \left\{ \begin{aligned} -\Delta \phi&=0,&\qquad&{\text {in }}\Omega , \\ \phi&= 1,&\qquad&{\text {on }}\Gamma _\text{epi}, \\ \phi&= 0,&\qquad&{\text {on }}\Gamma _\text{endo}, \\ \nabla \phi \cdot \textbf{n}&=0,&\qquad&{\text {on }}\partial \Omega \setminus (\Gamma _\text{endo} \cup \Gamma _\text{epi}). \end{aligned} \right. \end{aligned}$$
  Then, the transmural distance gradient $\nabla \phi$ is used to build the unit transmural direction:
  $$\begin{aligned} \widehat{\varvec{e}}_t=\frac{\nabla \phi }{\Vert \nabla \phi \Vert }. \end{aligned}$$
3. Normal direction: A normal (or apico-basal) direction $\varvec{k}$ (which is directed from the apex towards the base) is introduced and used to build the unit normal direction $\widehat{\varvec{e}}_n$:
  $$\begin{aligned} \widehat{\varvec{e}}_n = \frac{\varvec{k} - (\varvec{k} \cdot \widehat{\varvec{e}}_t )\widehat{\varvec{e}}_t}{\Vert \varvec{k} - (\varvec{k} \cdot \widehat{\varvec{e}}_t )\widehat{\varvec{e}}_t \Vert }. \end{aligned}$$
  The normal direction $\varvec{k}$ can be computed following one of these approaches (see also Fig. 5):

- Rossi-Lassila (RL) et al. approach [31]: $\varvec{k}$ is defined as the vector $\textbf{n}_\text{base}$, i.e. the outward normal to the basal plane, that is $\varvec{k}=\textbf{n}_\text{base}$.
- Bayer-Trayanova (BT) et al. approach [32]: $\varvec{k}$ is the gradient of the solution $\psi$ ($\varvec{k}=\nabla \psi$), which can be obtained by solving the following LD problem:
  2 $$\begin{aligned} \left\{ \begin{aligned} -\Delta \psi&=0,&\qquad&{\text {in }}\Omega , \\ \psi&= 1,&\qquad&{\text {on }}\Gamma _\text{base}, \\ \psi&= 0,&\qquad&{\text {on }}\Gamma _\text{apex}, \\ \nabla \psi \cdot \textbf{n}&=0,&\qquad&{\text {on }}\partial \Omega \setminus (\Gamma _\text{base} \cup \Gamma _\text{apex}). \end{aligned} \right. \end{aligned}$$
- Doste et al. approach [33]: $\varvec{k}$ is a weighted sum of the apico-basal ($\nabla \psi _\text{ab}$) and apico-outflow-tract ($\nabla \psi _\text{ot}$) directions, obtained using an interpolation function *w*:
  $$\begin{aligned} \varvec{k} = w\nabla \psi _\text{ab} + (1-w)\nabla \psi _\text{ot}, \end{aligned}$$
  where $\psi _\text{ab}$ and $\psi _\text{ot}$ are obtained by solving LD problems in the form of (2) where $\Gamma _\text{base}=\Gamma _\text{mv}$ (for $\psi _\text{ab}$) and $\Gamma _\text{base}=\Gamma _\text{av}$ (for $\psi _\text{ot}$), respectively. Moreover, the interpolation function *w* is obtained by solving:
  $$\begin{aligned} \left\{ \begin{aligned} -\Delta w&=0,&\qquad&{\text {in }}\Omega ,\\ w&= 1,&\qquad&{\text {on }}\Gamma _\text{mv} \cup \Gamma _\text{apex}, \\ w&= 0,&\qquad&{\text {on }}\Gamma _\text{av}, \\ \nabla w \cdot \textbf{n}&=0,&\qquad&{\text {on }}\partial \Omega \setminus (\Gamma _\text{av} \cup \Gamma _\text{mv} \cup \Gamma _\text{apex}). \end{aligned} \right. \end{aligned}$$
- Piersanti et al. approach [3]: for each point in $\Omega$, a unique normal direction $\varvec{k}$ is selected among the gradient of several normal directions $\varvec{k}=\nabla \psi _\text{i}$ ($\mathrm {i=ab,v,r}$), where $\psi _\text{i}$ are obtained by solving the following LD problem
  3 $$\begin{aligned} \left\{ \begin{aligned} -\Delta \psi _\text{i}&=0,&\qquad&{\text {in }}\Omega , \\ \psi _\text{i}&= \chi _\text{a},&\qquad&{\text {on }}\Gamma _\text{a}, \\ \psi _\text{i}&= \chi _\text{b},&\qquad&{\text {on }}\Gamma _\text{b}, \\ \nabla \psi _{i} \cdot \textbf{n}&=0,&\qquad&{\text {on }}\partial \Omega \setminus (\Gamma _\text{a} \cup \Gamma _\text{b}). \end{aligned} \right. \end{aligned}$$
  Please refer to [3] for further details about the selection procedure for $\varvec{k}$ and the specific choices of $\chi _\text{a}$, $\chi _\text{b}$, $\Gamma _\text{a}$ and $\Gamma _\text{b}$ in problem (2) made for $\psi _\text{i}$ ($i=\text{ab},\text{v},\text{r}$).

The BT approach is used in the ventricular and spherical slab geometry types, see also Fig. 5a. The BT and RL approaches can be adopted in the based ventricular geometry (by setting either Algorithm type equal to BT or RL in the parameter file, respectively), whereas the Doste approach is used in the complete ventricular geometry, see also Fig. 5b. Finally, the Piersanti approach is employed for the atrial geometry, see also Fig. 5c.

**Fig. 6:**
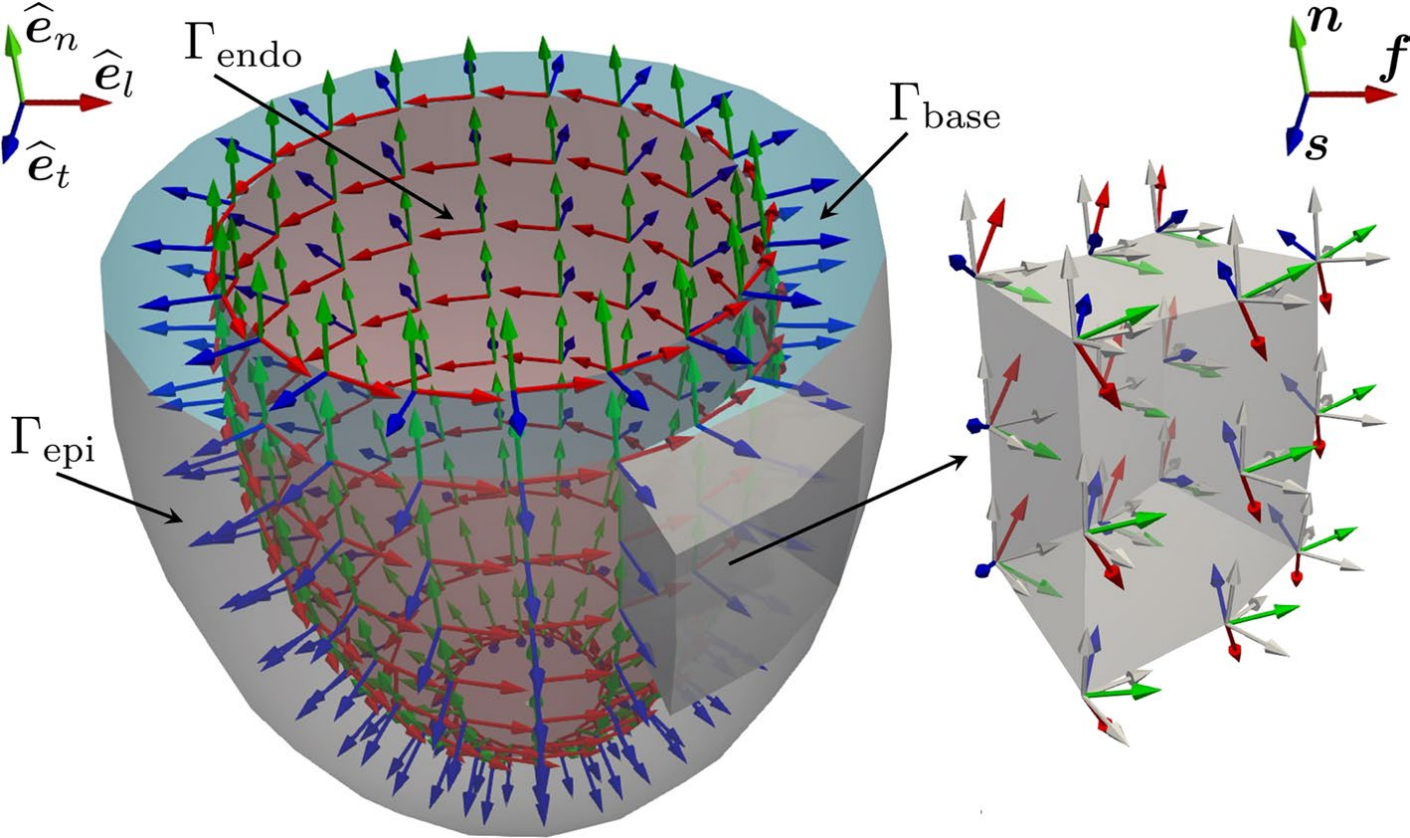
Representation of the local coordinate system employed by a LDRBM for an idealized ventricular domain. Only directions on the endocardium $\Gamma _\text{endo}$ are represented. In blue: unit transmural direction, $\widehat{\varvec{e}}_t$. In green: unit normal direction, $\widehat{\varvec{e}}_n$. In red: unit longitudinal direction, $\widehat{\varvec{e}}_l$. Right: zoom on a slab of the left ventricular myocardium showing the three final myofibers orientations ${\varvec{f}}$, ${\varvec{s}}$ and ${\varvec{n}}$

4. Local coordinate system: For each point of the domain an orthonormal local coordinate axial system is defined by $\widehat{\varvec{e}}_t$, $\widehat{\varvec{e}}_n$ and the unit longitudinal direction $\widehat{\varvec{e}}_l$ (orthogonal to the previous ones), as shown in Fig. 6:
  4 $$\begin{aligned} Q=\left[ \widehat{\varvec{e}}_l, \widehat{\varvec{e}}_n, \widehat{\varvec{e}}_t\right] = \left\{ \begin{aligned} \widehat{\varvec{e}}_t&= \frac{\nabla \phi }{\Vert \nabla \phi \Vert }, \\ \widehat{\varvec{e}}_n&= \frac{\varvec{k} - (\varvec{k} \cdot \widehat{\varvec{e}}_t )\widehat{\varvec{e}}_t}{\Vert \varvec{k} - (\varvec{k} \cdot \widehat{\varvec{e}}_t )\widehat{\varvec{e}}_t \Vert }, \\ \widehat{\varvec{e}}_l&= \widehat{\varvec{e}}_n \times \widehat{\varvec{e}}_t. \end{aligned} \right. \end{aligned}$$
5. Axis rotation: The reference frame is rotated with the purpose of defining the myofibers orientation: ${\varvec{f}}$ the fiber direction, ${\varvec{n}}$ the sheet-normal direction and ${\varvec{s}}$ the sheet direction. Specifically, $\widehat{\varvec{e}}_l$ rotates counter-clockwise around $\widehat{\varvec{e}}_t$ by the helical angle $\alpha$, whereas the transmural direction $\widehat{\varvec{e}}_t$ is rotated counter-clockwise around $\widehat{\varvec{e}}_l$ by the sheetlet angle $\beta$, see Fig. 6:
  $$\begin{aligned} \left[ \widehat{\varvec{e}}_l, \widehat{\varvec{e}}_n, \widehat{\varvec{e}}_t\right] \longrightarrow \left[ {\varvec{f}}, {\varvec{n}}, {\varvec{s}}\right] , \end{aligned}$$
  The rotation angles follow the linear relationships:
  $$\begin{aligned} \alpha (\phi ) = \alpha _\text{endo}(1-\phi )+\alpha _\text{epi}\phi , \qquad \beta (\phi ) = \beta _\text{endo}(1-\phi )+\beta _\text{epi}\phi , \end{aligned}$$
  where $\alpha _\text{endo}$, $\alpha _\text{epi}$, $\beta _\text{endo}$, $\beta _\text{epi}$ are suitable helical and sheetlet rotation angles on the epicardium and endocardium (specifying in the parameter file alpha epi, alpha endo, beta epi, beta endo). Moreover, for the complete ventricular geometry it is possible to set specific fiber and sheet angle rotations in the outflow tract (OT) region (i.e. around the aortic valve ring) by specifying alpha epi OT, alpha endo OT, beta epi OT, beta endo OT). Finally, for the atrial geometry type, no transmural variation in the myofibers direction is prescribed and the three unit directions correspond to the final myofibers directions $[\widehat{\varvec{e}}_l, \widehat{\varvec{e}}_n, \widehat{\varvec{e}}_t] =[{\varvec{f}}, {\varvec{n}}, {\varvec{s}}]$.

In order to represent the fiber architecture, LDRBMs use the gradient of specific intra-chamber distances, by means of harmonic problems, combined with a precise definition of boundary sections where boundary conditions are prescribed. This strategy makes the fibers less open to subjective variability. On the other hand, the myofiber orientations could be adapted to a patient-specific setting by simply changing the parameters involved in LDRBMs (e.g. the helical and sheetlet angles $\alpha$ and $\beta$). Therefore, unlike other RBMs requiring manual or semi-automatic interventions, LDRBMs can be easily applied to any arbitrary patient-specific geometry [3].

### Comparison to existing software

Several packages have been developed and are available in the framework of cardiac fibers generation.

Meshtools^7^ [43] is a comand-line tool designed to automate image-based mesh generation and manipulate tasks in cardiac modeling workflows, such as operations on label fields and/or geometric features; it integrates seamlessly with the openCARP^8^ ecosystem [44]; the algorithms supported are only for left ventricular geometries and of BT type. KIT-IBT-LDRB_Fibers^9^ is a MATLAB tool for generating left and bi-ventricular fibers; the original BT algorithm was adapted to eliminate a discontinuity in the fiber field in correspondence of the free walls and to yield a fiber rotation that is directly proportional to the transmural Laplace solution (approximately linear across the wall) [45]. CARDIO SUITE for GIMIAS^10^ includes tools for patient-specific modelling that allow to generate the FE meshes required for the simulations and to build additional structures such as fiber orientation [46]; at the time of writing and to the best of our knowledge, the latest version was released in 2016. SimCardio^11^ is advertised as the only fully open-source software package providing a complete pipeline from medical image data segmentation to patient specific blood flow simulation and analysis; its module svFSI supports specifying distributed fiber and sheet direction generated by BT-like rule-based algorithms.

Poisson interpolation algorithms [35] have inspired the implementation of fiber generation packages, despite being generally more computationaly demanding than, e.g., RL or BT algorithms [31]. This class of methods has been implemented in Cardiac Chaste,^12^ which supports automatic generation of mathematical model for fiber orientation associated with both idealized and anatomically-based geometry meshes [47], and in BeatIt,^13^ which is to our knowledge the only publicly available software which natively supports the generation of fiber architectures also for atrial geometries [48].

Compared to the software described above, the strengths of $\texttt {life}^{\texttt {x}}$-fiber reside in its user-friendly interface and in its generality, by supporting either idealized and realistic, (left) ventricular and atrial geometries and for each of them the user can selected one of the different state-of-the-art algorithms described in Section “Laplace-Dirichlet rule-based methods”. Finally, $\texttt {life}^{\texttt {x}}$-fiber offers a seamless integration with many other cardiac core models based on $\texttt {life}^{\texttt {x}}$ (such as electrophysiology, mechanics, electromechanics, and blood fluid dynamics) for modeling the cardiac function from single-chamber to whole-heart simulations which will be targeted by future releases.

## Implementation

In this section we introduce the technical specifications of $\texttt {life}^{\texttt {x}}$-fiber as well as a thorough documentation of the user interface exposed. The users will be guided from downloading it to running a full simulation of the algorithms presented in Section “Laplace-Dirichlet rule-based methods”

### Technical specifications

Here we specify the package content, copyright and licensing information and software and hardware specifications required by $\texttt {life}^{\texttt {x}}$-fiber.

#### Package content

$\texttt {life}^{\texttt {x}}$-fiber is shipped in binary form as an AppImage^14^ executable.

This provides a universal package for x86-64 Linux operating systems, without the need to deliver different distribution-specific versions. From the user’s perspective, this implies an effortless *download-then-run* process, without having to manually take care of installing the proper system dependencies required.

Once the source code will be made publicly accessible, a standard *build-from-source* procedure with automatic installers will be available to make the dependencies setup tailored to the specific hardware of HPC facilities or cloud platforms.

#### License and third-party software

This work is copyrighted by the $\texttt {life}^{\texttt {x}}$-fiber authors and licensed under the Creative Commons Attribution Non-Commercial No-Derivatives 4.0 International License.^15^

$\texttt {life}^{\texttt {x}}$-fiber makes use of third-party libraries. Please note that such libraries are copyrighted by their respective authors (independent of $\texttt {life}^{\texttt {x}}$ and $\texttt {life}^{\texttt {x}}$-fiber authors) and are covered by various permissive licenses.

The third-party software bundled with (in binary form), required by, copied, modified or explicitly used in $\texttt {life}^{\texttt {x}}$-fiber include the following packages [8]:

- $\texttt {life}^{\texttt {x}}$^16^: the open-source, high-performance framework providing the core functionalities for the numerical solution of the Finite Element problems described in Section “Laplace-Dirichlet rule-based methods”;
- Boost^17^: its modules Filesystem and Math are used for manipulating files/directories and for advanced mathematical functions and interpolators, respectively;
- deal.II^18^: it provides support to mesh handling, assembling and solving Finite Element problems (with a main support to third-party libraries as PETSc^19^ and Trilinos^20^ for linear algebra data structures and solvers) and to input/output functionalities;
- VTK^21^: it is used for importing external surface or volume input data and coefficients appearing in the mathematical formulation.

Some of the packages listed above, as stated by their respective authors, rely on additional third-party dependencies that may also be bundled (in binary form) with $\texttt {life}^{\texttt {x}}$-fiber, although not used directly. These dependencies include: ADOL-C,^22^ARPACK-NG,^23^BLACS,^24^Eigen,^25^FFTW,^26^GLPK,^27^HDF5,^28^HYPRE,^29^METIS,^30^MUMPS,^31^NetCDF,^32^OpenBLAS,^33^ParMETIS,^34^ScaLAPACK,^35^Scotch,^36^SuiteSparse,^37^SuperLU,^38^oneTBB,^39^p4est.^40^

The libraries listed above are all free software and, as such, they place few restrictions on their use. However, different terms may hold. Please refer to the content of the folder doc/licenses/ for more information on license and copyright statements for these packages.

Finally, an MPI installation (such as OpenMPI^41^ or MPICH^42^) may also be required to successfully run $\texttt {life}^{\texttt {x}}$ executables in parallel.

#### Software and hardware requirements

As an AppImage, $\texttt {life}^{\texttt {x}}$-fiber has been built on Debian Buster (the current oldstable version)^43^ following the *“Build on old systems, run on newer systems”* paradigm.^44^

Therefore, it is expected to run on (virtually) any *recent enough* x86-64 Linux distribution, assuming that glibc^45^ version 2.28 or higher is installed.

### Quick start guide: running the $\texttt {life}^{\texttt {x}}$-fiber executable

The following steps are required in order to run the $\texttt {life}^{\texttt {x}}$-fiber executable.

#### Download and installation

The $\texttt {life}^{\texttt {x}}$-fiber release archive can be downloaded from https://doi.org/10.5281/zenodo.5810268. After extracting the archive, the AppImage file should be made executable by typing the following command in a terminal:
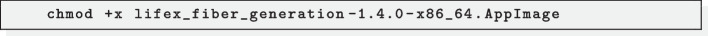


Finally, lifex_fiber_generation-1.4.0-x86_64.AppImage can be executed with:
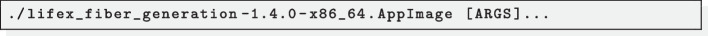


Root permissions are **not** required. Please note that, in order for the above procedure to succeed, AppImage relies upon the userspace filesystem framework FUSE^46^ which is assumed to be installed on your system. In case of errors, the following commands might be decisive:
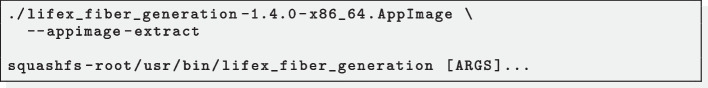


We also refer the reader to the AppImage troubleshooting guide.^47^

#### Step 0: parameter file configuration

Each $\texttt {life}^{\texttt {x}}$ application or example, including the $\texttt {life}^{\texttt {x}}$-fiber executable described in Section “Download and installation”, defines a set of parameters that are required in order to be run [8]. They involve problem-specific parameters (such as constitutive relations, geometry, time interval, boundary conditions) as well as numerical parameters (types of linear/non-linear solvers, tolerances, maximum number of iterations) or output-related options.

In case an application has sub-dependencies (such as a linear solver), also the related parameters are included (typically in a proper subsection).

Every application comes with a set of command line options, which can be printed using the -h (or –help) flag:
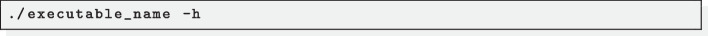


The first step before running an executable is to generate the parameter file(s) containing all the default parameter values. This is done via the -g (or –generate-params) flag:
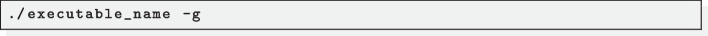


that by default generates a parameter file named after the executable, in .prm format.

By default, only parameters considered *standard* are printed. The parameter file verbosity can be decreased or increased by passing an optional flag minimal or full to the -g flag, respectively:
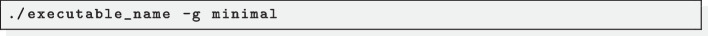


The parameter basename to generate can be customized with the -f (or –params-filename) option:
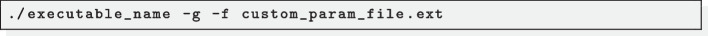


Absolute or relative paths can be specified.

At user’s option, in order to guarantee a flexible interface to external file processing tools, the parameter file extension ext can be chosen among three different interchangeable file formats prm, json or xml, from the most human-readable to the most machine-readable.

As an example, the three parameter files displayed in Listings 1, 2 and 3 are semantically equivalent.

We highlight that, following with the design of the ParameterHandler class of deal.II,^48^ each parameter is provided with:

- a given pattern, specifying the parameter type (e.g. boolean, integer, floating-point number, string, list, ...) and, whenever relevant, a range of admissible values (*pattern description*);
- a default value, printed in the parameter file upon generation and implicitly assumed if the user omits a custom value;
- the *actual* value, possibly overriding the default one;
- a documentation string;
- a global index.

All of these features, combined to a runtime check for the correctness of each parameter, make the code syntactically and semantically robust with respect to possible errors or typos introduced unintentionally.
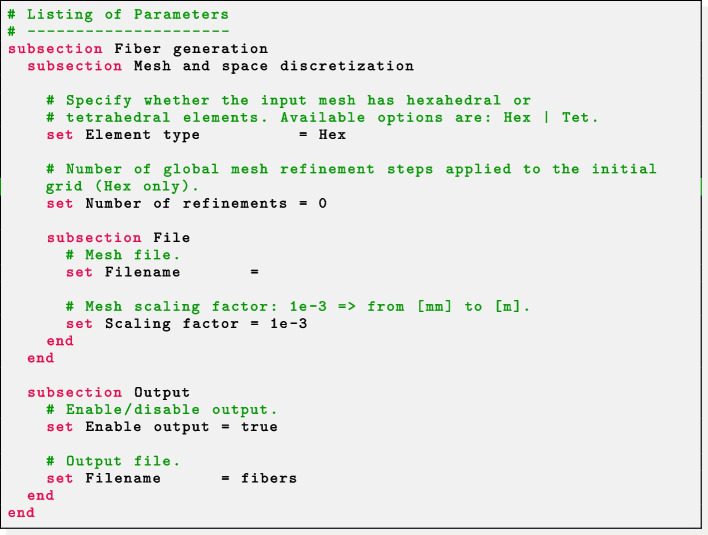

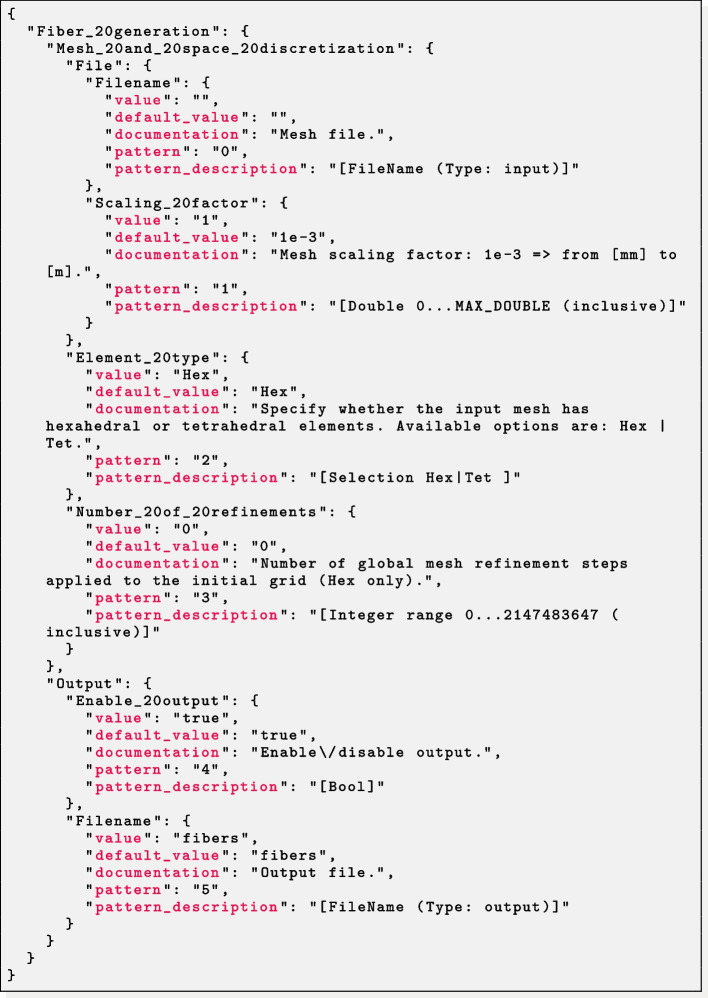

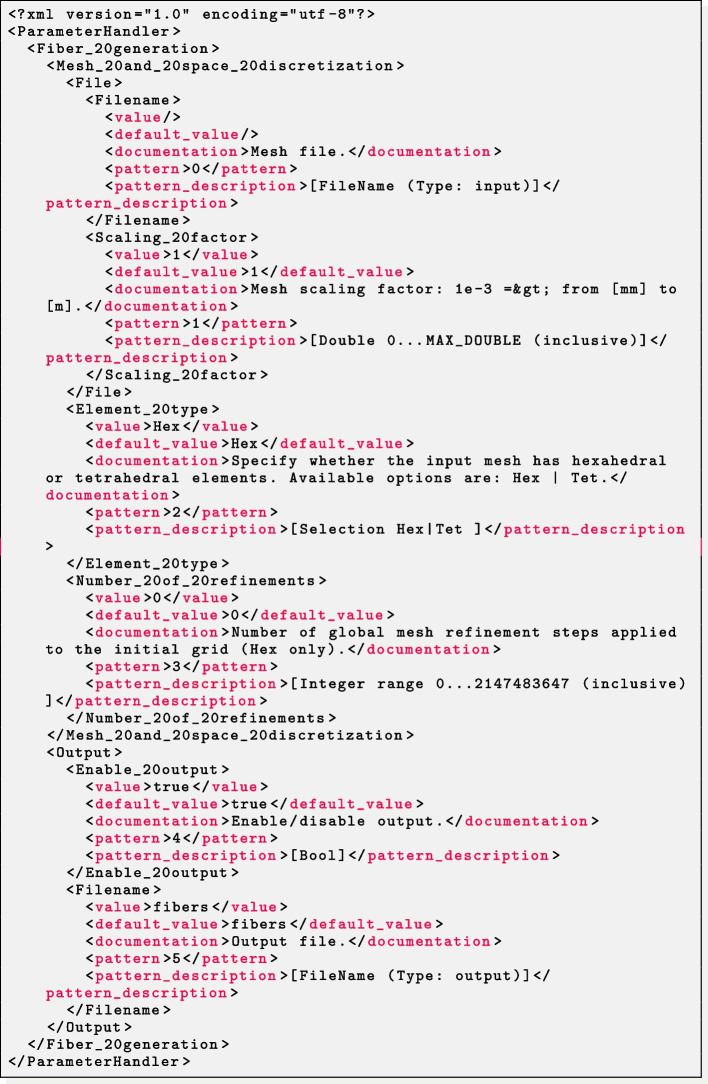


Once generated, the user can modify, copy, move or rename the parameter file depending on their needs.

#### Step 1: run

The executable can be run by simply omitting the -g, whereas the -f option is used to specify the parameter file to be *read* (as opposed to *written*, in generation mode), e.g.:
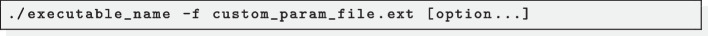


If no -f flag is provided, a file named executable_name.prm is assumed to be available in the directory where the executable is run from.

The path to the directory where all the app output files will be saved to can be selected via the -o (or –output-directory) flag:
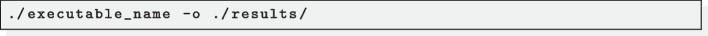


If the specified directory does not already exist, it will be created. By default, the current working directory is used.

Absolute or relative paths can be specified for both the input parameter file and the output directory.

*Parallel run* In order to run an app in parallel, the mpirun or mpiexec wrapper commands (which may vary depending on the MPI implementation available on your machine) should be prepended, e.g.:
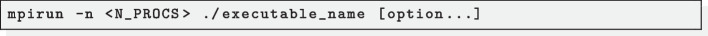


where <N_PROCS> is the desired number of parallel processes to run on.

As a rule of thumb, 10,000 to 100,000 degrees of freedom per process should lead to the best performance.

*Dry run and parameter file conversion* Upon running, a parameter log file is automatically generated in the output directory, that can be used later to retrieve which parameters had been used for a specific run.

By default, log_params.ext will be used as its filename. This can be changed via the -l (or –log-file) flag, e.g.:
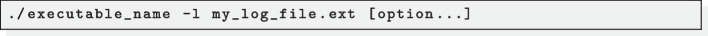


The file extension is not mandatory: if unspecified, the same extension as the input parameter file will be used.

If the **dry run** option is enabled via the -d (or –dry-run) flag, the execution terminates right after the parameter log file generation. This has a two-fold purpose:

1. checking the correctness of the parameters being declared and parsed *before* running the actual simulation (if any of the parameters does not match the specified pattern or has a wrong name or has not been declared in a given subsection then a runtime exception is thrown);
2. **converting** a parameter file between two different formats/extensions. For example, the following command converts input.xml to
  output.json:

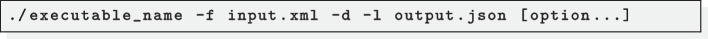

## Results and discussion

The LDRBMs described in Section “Laplace-Dirichlet rule-based methods” have been applied to a set of idealized or realistic test cases, namely ventricular and spherical slabs, based and complete left ventricular and left atrial geometries.

This section presents the results obtained, as well as a possible pipeline for reproducing such test cases, consisting of the following steps:

1. setting up input data (e.g. generating or importing computational meshes);
2. setting up the parameter files associated with a given simulation scenario and running the corresponding simulation;
3. post-processing the solution and visualizing the output.

A mesh sensitivity analysis has been performed and reported in Section “Mesh sensitivity and validation”.

### Input data

Additional input data (scripts, meshes and parameter files) associated with the guided examples described below can be downloaded from the release archive https://doi.org/10.5281/zenodo.5810268.

In this *getting started* guide, we provide different ready-to-use meshes, namely

- a set of four idealized geometries consisting of a ventricular slab, a spherical slab, an idealized based left ventricle and an idealized left atrium, see Fig. 4a–d;
- two realistic geometries composed by a left ventricle and a left atrium, see Fig. 4e, f.

The idealized meshes have been generated using the built-in CAD engine of gmsh, an open-source 3D FE mesh generator, starting from the corresponding gmsh geometrical models (represented by *.geo files, also provided) defined using their boundary representation, where a volume is bounded by a set of surfaces. For details about the geometrical definition of a model geometry we refer to the online documentation of gmsh.^49^

In order to perform the mesh generation, starting from the geometrical files provided in this tutorial, the following command can be run in a terminal:
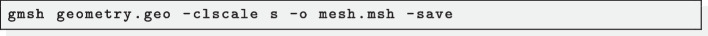


where geometry.geo is the geometrical file model, mesh.msh is the output mesh file, which will be provided as an input to the $\texttt {life}^{\texttt {x}}$-fiber app, and s $\in (0,1]$ is the mesh element size factor. To produce a coarser (finer) mesh the clscale factor can be reduced (increased).

The realistic left ventricle and left atrium have been produced starting from the open-source meshes adopted in [38] (for the left atrium^50^) and in [49] (for the left ventricle^51^) and using the Vascular Modelling Toolkit (vmtk) software [50] along with the semi-automatic meshing tools^52^ recently proposed in [51].

All the characteristic informations of the ready-to-use meshes, above described, are reported in Table 1.

**Table 1:** Mesh information regarding the ready-to-use meshes presented in this work. In particular the mesh quality is computed selecting the edge ratio option in the ParaView mesh quality filter

Geometry	Type	h_min_ [mm]	h_avg_ [mm]	h_max_ [mm]	#elements	#vertices	Quality
Ventricular slab	Hex	4.74	5.95	6.73	988	1400	1.69
Ventricular slab	Tet	3.10	6.76	8.71	2439	762	2.91
Spherical slab	Tet	1.96	3.46	4.70	18455	4924	2.46
Idealized left atrium	Tet	2.78	3.43	4.15	10039	3387	2.36
Idealized left ventricle	Tet	1.71	3.53	4.93	74488	16567	3.27
Realistic left ventricle	Tet	0.51	1.17	1.67	1885227	330703	2.80
Realistic left atrium	Tet	0.15	1.00	1.76	112994	33297	4.23

### Generating fibers

Parameter files for fiber generation are characterized by a common section named Mesh and space discretization. Select Element type = Tet for tetrahedral meshes or Element type = Hex for hexahedral meshes. Finally, specify in FE space degree the degree of the (piecewise continuous) polynomial FE space used to solve the LD problems described above. Finally, we remark that $\texttt {life}^{\texttt {x}}$-fiber internally treats all physical quantities as if they are provided in International System of Units (SI): therefore, a Scaling factor can be set in order to convert the input mesh from a given unit of measurement (e.g. if the mesh coordinates are provided in millimeters then Scaling factor must be set equal to 1e-3).

The Geometry type parameters enables to specify the kind of geometry provided in input, in order to apply the proper LDRBM algorithm among the ones described above. Once specified, parameters related to the specific algorithm and geometry will be parsed from a subsection named after the value of Geometry type.

All parameters missing from the parameter file will take their default value, which is hard-coded.
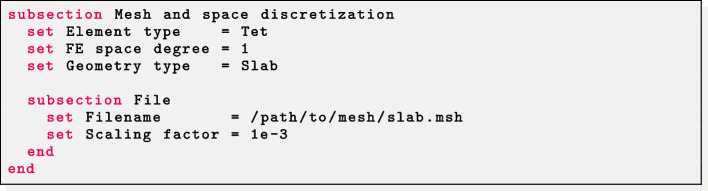


#### Slab fibers

The parameter Geometry type = Slab must be set to prescribe fibers in slab geometries and the path of the input mesh file in Filename.

*Ventricular slab* For a ventricular slab geometry the user should set Sphere slab = false. Labels for the top (Tags base up) and bottom (Tags base down) surfaces of the slab, as well as for the epicardium (Tags epi) and endocardium (Tags endo) must be prescribed. Finally, the helical and sheetlet rotation angles at the epicardium and endocardium must be provided in the corresponding alpha epi, alpha endo, beta epi, beta endo parameters.
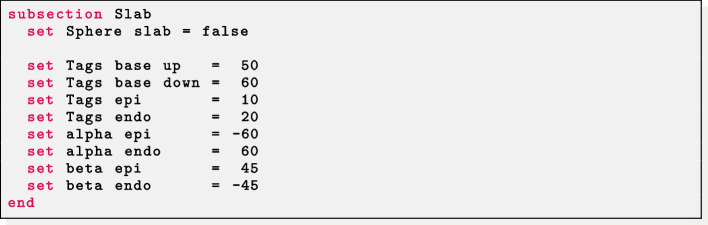


*Spherical slab* For a spherical slab geometry the user should set Sphere slab = true. The epicardial coordinates (*x*, *y*, *z*) of the north (North pole) and south (South pole) poles of the sphere of the slab, and the labels of the endocardium (Tags endo) and epicardium (Tags epi) must be prescribed. Finally, the helical and sheetlet rotation angles at the epicardium and endocardium must be provided in the alpha epi, alpha endo, beta epi, beta endo parameters. A fiber architecture for the sphere slab with radial fiber $\varvec{f}$ can be prescribed by setting in the parameter file Sphere with radial fibers = true. This consists of exchanging the sheet direction $\varvec{s}$ with the fiber direction $\varvec{f}$. Instead, a tangential (to the epicardial and endocardial surfaces) fiber field $\varvec{f}$ is assigned when Sphere with radial fibers = false.
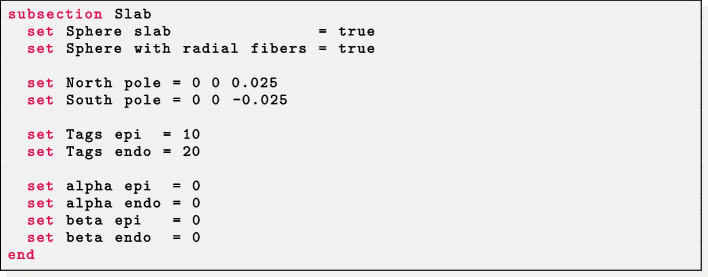


#### Left ventricular fibers

The parameter Geometry type = Left ventricle (Left ventricle complete) prescribes fibers in a based (complete) left ventricular geometry. Other mesh-related parameters have the same meaning as described above.
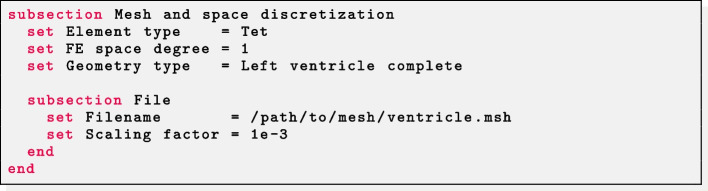


*Based left ventricle* The parameters needed are the labels for the basal plane (Tags base), the epicardium (Tags epi) and endocardium (Tags endo) of the ventricle. The RL or BT approach can be toggled via Algorithm type. The helical and sheetlet rotation angles at the epicardium and endocardium must be prescribed in alpha epi, alpha endo, beta epi, beta endo. Finally, for the RL approach the outward normal vector to the basal plane must be specified in Normal to base, whereas for the BT approach the apex epicardial coordinates (*x*, *y*, *z*) (Apex) of the ventricle is needed.
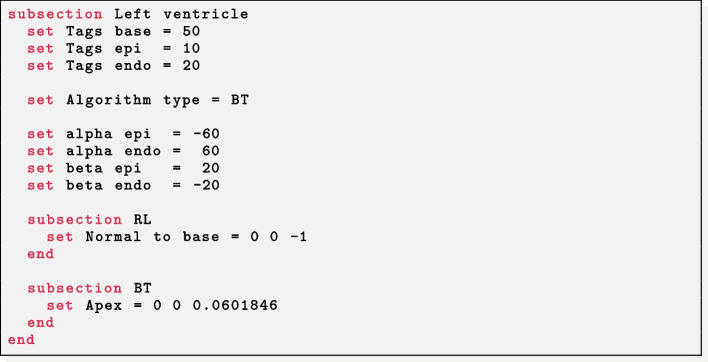


Selecting the RL approach as Algorithm type, this setup can be also exploited in bi-ventricular geometries (not included in the example meshes). In this case, the mesh must have two additional surface labels in the right ventricular endocardium: one delimiting the part facing to the septum (e.g. 15) and the other for the remaining part (e.g. 25). In this way, it is sufficient to set Tags endo as the two endocardial labels (excluding the right septum) and Tags epi as the epicardial label and the right endocardial septum label. Further detail on this approach can be found in [3].
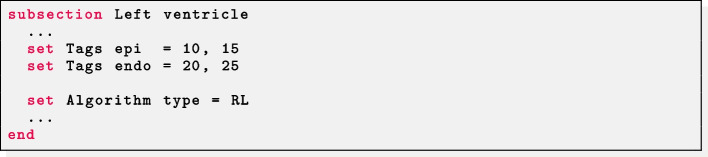


*Complete left ventricle* The labels for the mitral (Tags MV) and aortic (Tags AV) valve rings, the epicardium (Tags epi) and endocardium (Tags endo) of the ventricle are required. The apex epicardial coordinates (*x*, *y*, *z*) must be set in Apex. Finally, the helical and sheetlet rotation angles at the epicardium and endocardium must be prescribed in alpha epi, alpha endo, beta epi, beta endo. A specific helical and sheetlet rotation angles around the outflow tract of the left ventricle (i.e. the mitral valve ring) can be specified by setting alpha epi OT, alpha endo OT, beta epi OT, beta endo OT.
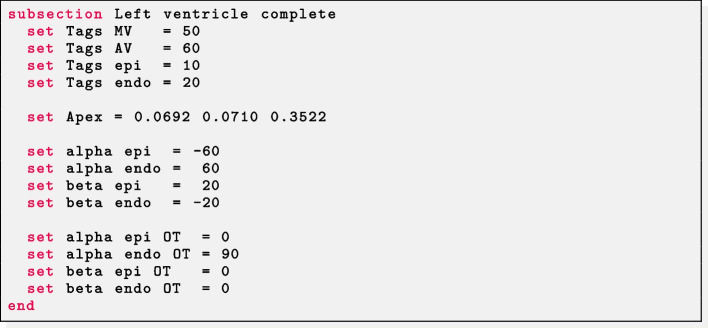


#### Left atrial fibers

Fibers in a left atrial geometry can be generated by setting Geometry type = Left atrium. Other mesh-related parameters have the same meaning as described above.
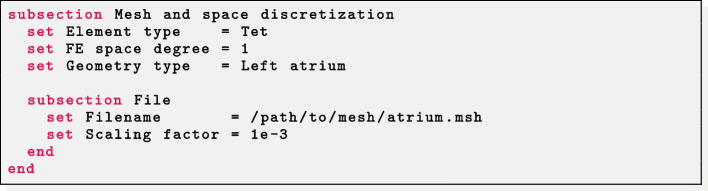


*Idealized left atrium* The parameter Appendage = false prescribes fibers in the hollow sphere geometry. The labels for the mitral valve ring (Tags MV), the right (Tags RPV) and left (Tags LPV) pulmonary veins rings, the epicardium (Tags epi) and endocardium (Tags endo) of the idealized atrium must be provided. Finally, the dimension of each atrial bundle is needed: Tau bundle MV for the mitral valve bundle; Tau bundle LPV and Tau bundle RPV for the left and right pulmonary valves ring bundles.
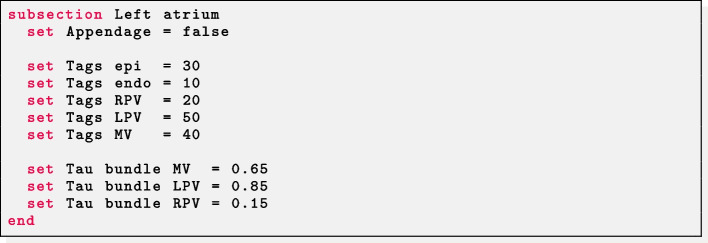


*Realistic left atrium* The parameter Appendage = true prescribes fibers in a realistic left atrial geometry. The user should provide labels for the mitral valve ring (Tags MV), the right (Tags RPV) and left (Tags LPV) pulmonary veins rings, the epicardium (Tags epi) and endocardium (Tags endo) of the idealized atrium. The epicardial coordinates (*x*, *y*, *z*) for the apex of the atrial appendage must be provided in Apex. Finally, the dimension of each atrial bundle is needed: for the mitral valve bundle Tau bundle MV; for the left and right pulmonary valves rings bundle Tau bundle LPV and Tau bundle RPV, respectively.
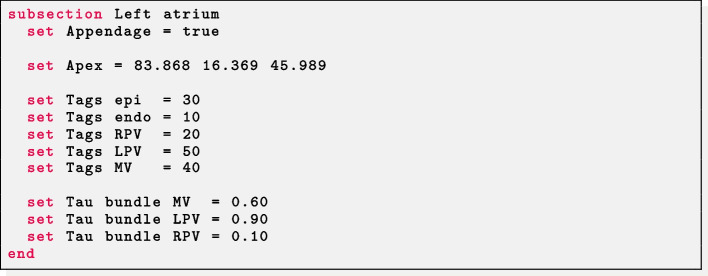


### Output and visualization

**Fig. 7:**
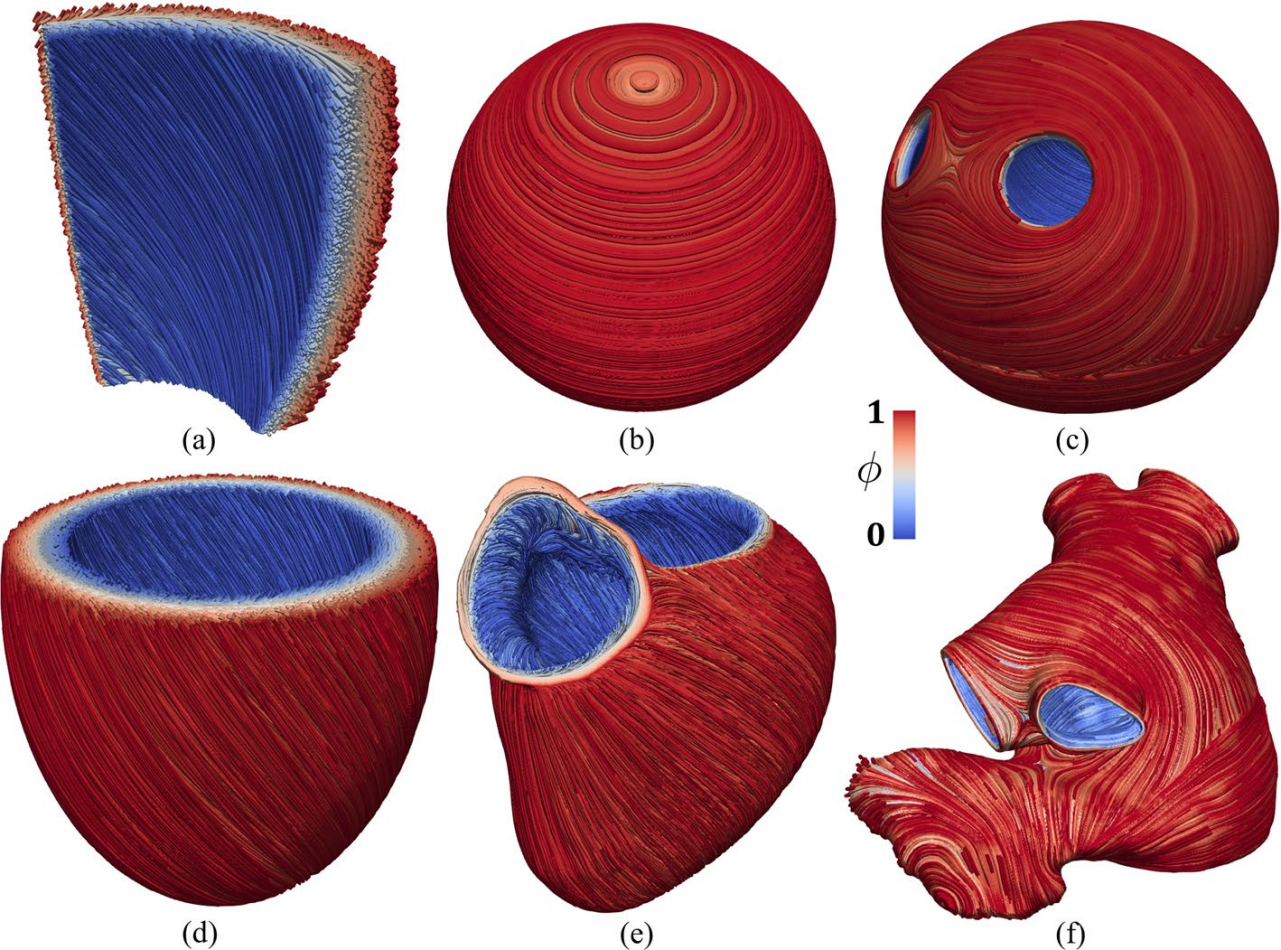
Fiber field $\varvec{f}$ visualized as streamlines. **a** Ventricular slab. **b** Spherical slab with circumferential fibers. **c** Idealized left atrium. **d** Idealized based left ventricle. **e** Realistic complete left ventricle. **f** Realistic left atrium

To enable the output the user should set Enable output = true and specify the corresponding output filename in Filename. This will produce a XDMF schema file named output_filename.xdmf (wrapped around a same-named HDF5 output file output_filename.h5) that can be visualized in ParaView,^53^ an open-source multi-platform data analysis and visualization application. Specifically, the streamtracer and the tube filters of ParaView can be applied in sequence to visualize the fiber fields, such as the one shown in Fig. 7.

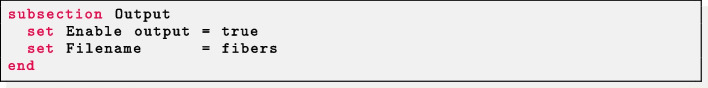



Moreover, the HDF5 file format guarantees that the output can easily be further post-processed, not only for visualization purposes but rather to be fed as an input to more sophisticated computational pipelines.

### Mesh sensitivity and validation

The robustness of the algorithms presented above is confirmed by performing a mesh sensitivity analysis. Specifically, we consider the ventricular slab geometry shown in Fig. 4a and two related sets of discretization with hexahedral and with tetrahedral elements, respectively. For each of the two element types, we run the fiber generation algorithm (see Section “Ventricular slab”) for four decreasing mesh sizes $h_1> h_2> h_3 > h_4$.

As the fibers field is orthonormalized, the difference between two numerical solutions is only due to the orientation angle. This allows us to define an error estimate as:

$$\begin{aligned} \Delta _\theta ^{i} = \left| \arccos \left( \textbf{f}_0^i \cdot \textbf{f}_0^4\right) \right| ,\ i=1,2,3, \end{aligned}$$

where $\textbf{f}_0^i$ is the fiber field computed on the mesh with size $h_i$ and the results on the finest mesh with size $h_4$ are considered as a reference solution.

**Table 2:** Mesh sensitivity analysis for hexahedral elements

i	*h* [mm]	#dofs	$\displaystyle \mathop {\text{avg}}\limits _\Omega \Delta _{\theta }^{i}\,[{}^\circ ]$	$\displaystyle \max _\Omega \Delta _{\theta }^{i}\,[{}^\circ ]$
1	5.95	1400	0.62	6.33
2	3.00	9477	0.24	4.54
3	1.50	69377	0.08	2.40
4	0.75	530145	–	–

**Table 3:** Mesh sensitivity analysis for tetrahedral elements

i	*h* [mm]	#dofs	$\displaystyle \mathop {\text{avg}}\limits _\Omega \Delta _{\theta }^{i}\,[{}^\circ ]$	$\displaystyle \max _\Omega \Delta _{\theta }^{i}\,[{}^\circ ]$
1	6.76	762	1.35	7.85
2	3.53	3989	0.50	5.57
3	1.76	25661	0.19	4.28
4	0.88	179338	–	–

**Fig. 8:**
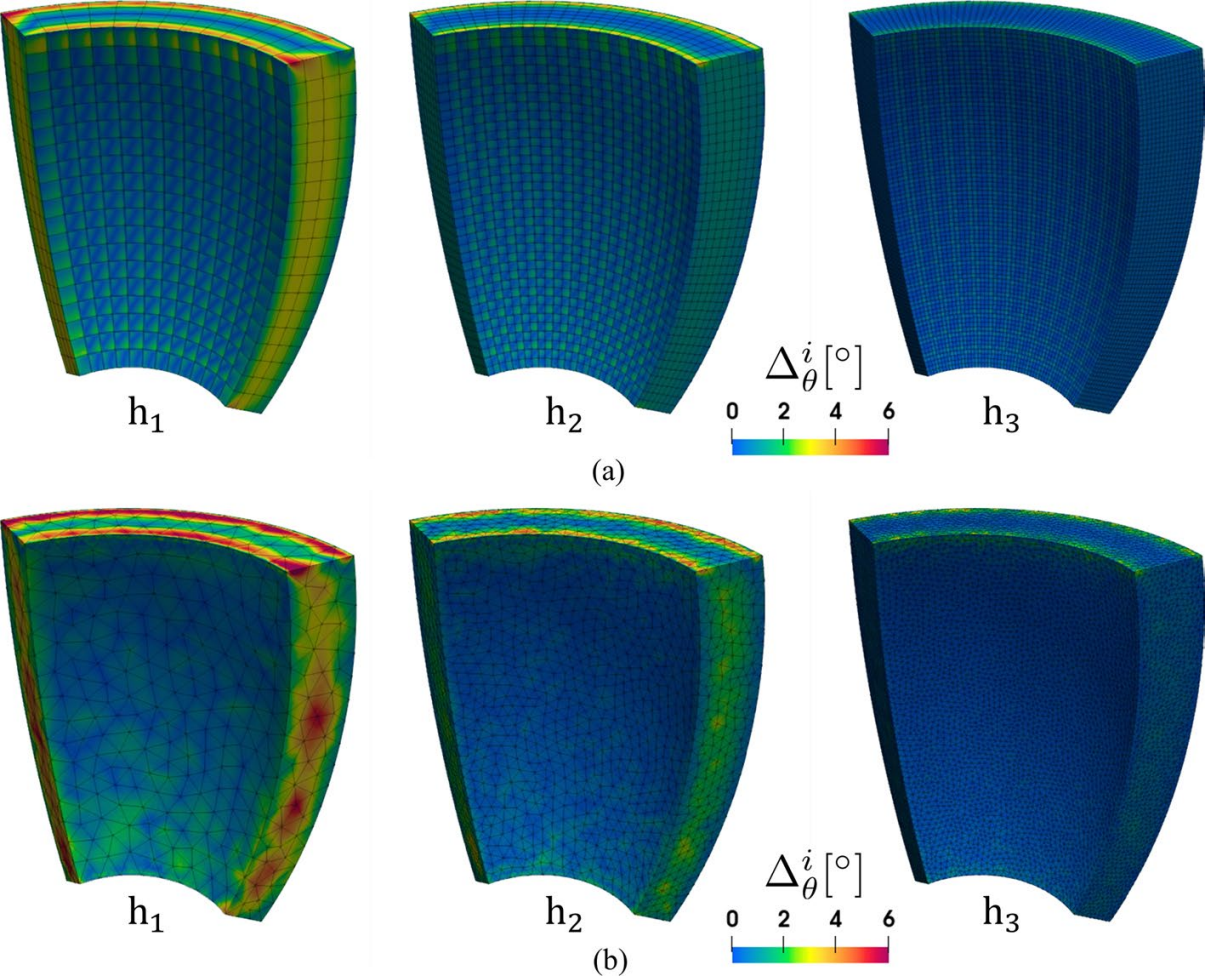
Mesh sensitivity analysis performed on the ventricular slab geometry and three different mesh sizes $h_1,h_2,h_3$. **a** Hexahedral meshes. **b** Tetrahedral meshes

We show the error distribution in the whole domain in Fig. 8, whereas Tables 2 and 3 report the average and maximum errors for the hexahedral and the tetrahedral meshes, respectively. We determine both an average and a maximum error smaller than 8${}^\circ$ even for the coarsest mesh. These values are very small and, in particular, much smaller than the physiological fibers dispersion angle [52].

The interest of accurately reconstructing a fiber orientation field consists of providing it as an input to more sophisticated computational models, such as for cardiac electrophysiology and electromechanics. As the dynamics of such physical models demands for a high resolution in both time and space [3, 17, 53], the LDRBM algorithms are typically run on fine meshes. Therefore, the impact of possible numerical errors due to the mesh size is to be considered negligible, as confirmed by the small errors presented above.

The proposed LDRBMs have been validated in [3], where the fiber orientation field computed numerically provided a satisfactory match to histological data.

Furthermore, the anisotropic nature of the fiber orientation strongly influences the electrophysiological, mechanical, and electromechanical cardiac function. Therefore, an indirect way to validate the fiber field provided by LDRBMs is to compute quantitative indices or biomarkers in such kind of simulations.

Matching quantitative indices in a physiological range is only possible when the fiber reconstruction algorithm is accurate enough: this is shown in [3] for the whole-heart electrophysiology, in [17] for the ventricular electromechanics, and in [53] for the whole-heart electromechanics.

### Future developments

As anticipated in Section ”Background”, $\texttt {life}^{\texttt {x}}$ has served as the core framework for the development of several *heart modules* for the simulation of cardiac electrophysiology, mechanics, electromechanics, and blood fluid dynamics models.

In the near future, the deployment of $\texttt {life}^{\texttt {x}}$ modules will follow two lines:

- more modules will be successively published in binary form, starting from an advanced solver for cardiac electrophysiology, blood fluid dynamics and other solvers for the cardiac function;
- in the meantime, the source code associated with previous binary releases will be gradually made publicly available under an open-source license.

In the long run, also modules unrelated to the cardiac function are expected to be included within $\texttt {life}^{\texttt {x}}$.

## Conclusions

$\texttt {life}^{\texttt {x}}$ is intended to provide the scientific community with an integrated FE framework for exploring many physiological and pathological scenarios using *in silico* experiments for the whole-heart cardiac function, boosting both the user and developer experience without sacrificing its computational efficiency and universality.

We believe that the release of $\texttt {life}^{\texttt {x}}$-fiber provides the scientific community with an invaluable tool for *in silico* scenario analyses of myofibers orientation; such a tool supports either idealized and realistic, (left) ventricular and atrial geometries. It also offers a seamless integration of LDRBMs into more sophisticated computational pipelines involving other core models – such as electrophysiology, mechanics and electromechanics – for the cardiac function, in a wide range of settings covering from single-chamber to whole-heart simulations.

The content of this initial release is published on the official website https://lifex.gitlab.io/heart.html: we encourage users to interact with the $\texttt {life}^{\texttt {x}}$ development community via the issue tracker^54^ of our public website repository. Any curiosity, question, bug report or suggestion is welcome.

News and announcements about $\texttt {life}^{\texttt {x}}$ will be posted to the official website https://lifex.gitlab.io/.

## Availability and requirements

- **Project name**: $\texttt {life}^{\texttt {x}}$-fiber
- **Project home page**: https://lifex.gitlab.io/heart.html
- **Operating system(s)**: Linux (x86-64)
- **Programming language**: C++
- **Other requirements**: glibc version 2.28 or higher
- **License**: CC BY-NC-ND 4.0
- **Any restrictions to use by non-academics**: no additional restriction.

## Data Availability

All input data, meshes and the binary executable of $\texttt {life}^{\texttt {x}}$ can be found at https://doi.org/10.5281/zenodo.5810268.

## Abbreviations

FE: Finite element
BT: Bayer-Trayanova
RL: Rossi-Lassila
HPC: High performance computing
LD: Laplace-Dirichlet
RBM: Rule-based method
LDRBM: Laplace-Dirichlet rule-based method
SI: International system of units

## Footnotes

1 https://www.dealii.org/.

2 http://gmsh.info.

3 https://ngsolve.org/.

4 http://www.vmtk.org.

5 https://bitbucket.org/aneic/meshtool/src/master/.

6 https://pypi.org/project/meshio/.

7 https://bitbucket.org/aneic/meshtool/src/master.

8 https://opencarp.org/.

9 https://github.com/KIT-IBT/LDRB_Fibers.

10 http://www.gimias.org/index.php/clinical-prototypes/cardiosuite.

11 http://simvascular.github.io/index.html.

12 https://www.cs.ox.ac.uk/chaste/cardiac_index.html.

13 https://github.com/rossisimone/beatit.

14 https://appimage.org/.

15 http://creativecommons.org/licenses/by-nc-nd/4.0/.

16 https://lifex.gitlab.io/.

17 https://www.boost.org/.

18 https://www.dealii.org/.

19 https://www.mcs.anl.gov/petsc/.

20 https://trilinos.github.io/.

21 https://vtk.org/.

22 https://github.com/coin-or/ADOL-C.

23 https://github.com/opencollab/arpack-ng.

24 https://www.netlib.org/blacs/.

25 https://eigen.tuxfamily.org/.

26 https://www.fftw.org/.

27 https://www.gnu.org/software/glpk/.

28 https://www.hdfgroup.org/solutions/hdf5/.

29 https://www.llnl.gov/casc/hypre/.

30 http://glaros.dtc.umn.edu/gkhome/metis/metis/overview.

31 http://mumps.enseeiht.fr/index.php?page=home.

32 https://www.unidata.ucar.edu/software/netcdf/.

33 https://www.openblas.net/.

34 http://glaros.dtc.umn.edu/gkhome/metis/parmetis/overview.

35 https://www.netlib.org/scalapack/.

36 https://gitlab.inria.fr/scotch/scotch.

37 https://people.engr.tamu.edu/davis/suitesparse.html.

38 https://portal.nersc.gov/project/sparse/superlu/.

39 https://oneapi-src.github.io/oneTBB/.

40 https://www.p4est.org/.

41 https://www.open-mpi.org/.

42 https://www.mpich.org/.

43 https://www.debian.org/releases/.

44 https://docs.appimage.org/introduction/concepts.html.

45 https://www.gnu.org/software/libc/.

46 https://www.kernel.org/doc/html/latest/filesystems/fuse.html.

47 https://docs.appimage.org/user-guide/troubleshooting/fuse.html.

48 https://www.dealii.org/current/doxygen/deal.II/classParameterHandler.html.

49 https://gmsh.info/doc/texinfo/gmsh.html.

50 https://doi.org/10.18742/RDM01-289.

51 https://doi.org/10.5281/zenodo.3890034.

52 https://github.com/marco-fedele/vmtk.

53 https://www.paraview.org.

54 https://gitlab.com/lifex/lifex.gitlab.io/-/issues.
